# Dose-Dependent Impact of Bee Pollen Supplementation on Macroscopic and Microscopic Structure of Femoral Bone in Rats

**DOI:** 10.3390/ani11051265

**Published:** 2021-04-28

**Authors:** Monika Martiniakova, Ivana Bobonova, Robert Toman, Branislav Galik, Maria Bauerova, Radoslav Omelka

**Affiliations:** 1 Department of Zoology and Anthropology, Faculty of Natural Sciences, Constantine the Philosopher University in Nitra, 949 74 Nitra, Slovakia; 2 Department of Botany and Genetics, Faculty of Natural Sciences, Constantine the Philosopher University in Nitra, 949 74 Nitra, Slovakia; ivana.bobonova@gmail.com (I.B.); mbauerova@ukf.sk (M.B.); 3 Department of Veterinary Sciences, Faculty of Agrobiology and Food Resources, Slovak University of Agriculture in Nitra, 949 76 Nitra, Slovakia; robert.toman@uniag.sk; 4 Department of Animal Nutrition, Faculty of Agrobiology and Food Resources, Slovak University of Agriculture in Nitra, 949 76 Nitra, Slovakia; branislav.galik@uniag.sk

**Keywords:** bee pollen, nutrition, bone structure, bone health, rat

## Abstract

**Simple Summary:**

Bee pollen is considered an interesting feed supplement with beneficial health impacts. It contains many basic nutritional compounds that improve growth performance, development and immune response of animals. However, its effect on bone structure has been studied to a limited extent and the results published so far are ambiguous. Therefore, the impact of bee pollen supplementation on selected bone characteristics of rats was investigated in our study. We determined a dose-dependent effect of bee pollen administration on macroscopic and microscopic structure of femoral bone. Several negative effects of bee pollen supplementation at the level of 0.75% on bone features have been demonstrated, while the level of 0.5% did not influence these properties in rats.

**Abstract:**

Bee pollen has been successfully used as a feed additive with beneficial impacts on productive, reproductive, and immune conditions of animals. However, its effect on bone structure and bone health remains controversial. Therefore, the purpose of our study was to examine the impact of bee pollen supplementation on macroscopic and microscopic structure of a femoral bone using rats as suitable animal models. Male rats (1 month-old) were assigned into three groups: control (C group) that was fed a standard diet without bee pollen and two bee pollen supplemented groups (P1 and P2 groups) that received an experimental diet including 0.5% and 0.75% of bee pollen, respectively, for 3 months. A number of unfavorable effects of 0.75% bee pollen administration on bone weight, cortical bone thickness, calcium content, alkaline phosphatase activity, sizes of primary osteons’ vascular canals, Haversian canals and secondary osteons in the cortical bone have been recorded, whereas these bone parameters were significantly decreased in the P2 group versus the C group. On the contrary, the concentration of 0.5% did not affect any of bone features mentioned above. In conclusion, the impact of bee pollen supplementation on femoral bone structure of rats depends on the dose used.

## 1. Introduction

Bee pollen is a conglomerate of flower pollen collected by the bees and mixed with both honeybee salivary enzymes and nectar [1]. It is considered a well-known feed supplement. This natural product is rich in proteins, reducing sugars, lipids, essential amino acids, fatty acids, minerals, vitamins, phytosterols, and flavonoids [2,3]. Aforementioned components are responsible for its high nutritional value [4]. Due to the fact that other bioactive compounds are also represented (e.g., carotenoids, bioactive peptides, organic acids), having beneficial health effects, it is a useful tool for therapeutic approach as well. Favorable health impacts of bee pollen are the result of the presence of polyphenol compounds with antioxidant activity, phytosterols and polyunsaturated fatty acids with antitumor properties, as well as flavonoid glycosides with increased immunological activity [2,5]. It is also regarded as a promising anti-microbial agent [3]. Bee pollen supplementation enhanced growth performance, carcass quality, and immunity of farm animals [2]. However, its effect on bone macroscopic and microscopic structure remains controversial. Yamaguchi et al. [6] revealed a higher calcium (Ca) concentration in the femoral bone of rats treated with bee pollen extract (5 and 10 mg/100 g body weight). No effect of dietary bee pollen inclusion (0.5% and 1.0% of feed) on Ca content and bone mineralization has been observed in broiler chickens [7,8]. Similarly, the concentration of Ca was not affected by bee pollen supplementation at the level of 0.2% in rats [9]. On the contrary, Tomaszewska et al. [1] reported a decreased bone mineralization, bone length, bone weight, and mean relative wall thickness in Japanese quails receiving a bee pollen dietary supplemented diet (1.0%). Bobonova et al. [10] also indicated a reduced bone weight in female rats fed with a bee pollen supplemented diet at the level of 0.75%.

Due to ambiguous results published so far, the purpose of this study was to examine the impact of bee pollen supplementation at two doses (0.5% and 0.75% of feed) on selected macroscopic and microscopic properties of femoral bone using male rats as suitable animal model.

## 2. Materials and Methods

One-month-old male Wistar rats (n = 15) were bred at the Slovak University of Agriculture in Nitra. They were individually housed in plastic cages (Techniplast, Italy) under standardized conditions with 12:12 h light-dark regime, 55 ± 10% of humidity and temperature of 22 ± 2 °C. Complete granular diet for rats (Bonagro, Czech Republic) and water were supplied on *ad libitum* basis. The rats were assigned into three groups of five animals each: control (C group) was fed standard complete diet without bee pollen and two bee pollen groups (P1 and P2 groups) received experimental diet including 0.5% and 0.75% of bee pollen, respectively. Dried rape (*Brassica napus L.*) bee pollen was obtained from local beekeepers from western Slovakia. Nutrient composition of bee pollen (in g/kg of dry matter) was as follows: organic matter 429.2, crude proteins 190.9, crude fat 29.5, crude fiber 13.4, calcium 1.8, and phosphorus 5.3. The experiment lasted for 3 months, identical to our previous studies [9,10]. The doses used were selected according to the studies by Yamaguchi et al. [6] and Bobonova et al. [10].

After euthanasia of rats, blood samples were collected and plasma alanine aminotransferase (ALT), aspartate aminotransferase (AST) and alkaline phosphatase (ALP) were assayed by commercially available kits (Stamar, Poland). Subsequently, both femoral bones (n = 30) were dissected, weighed, and their lengths were measured. The total body weight of rats from all groups was also recorded.

Thin sections for histological analysis of cortical bone tissue were prepared using special microtome (Leica, Germany) according to Martiniakova et al. [9]. They were evaluated by reputable classification systems [11,12]. Measured parameters included area of primary osteons’ vascular canals (n = 398), Haversian canals (n = 323), and secondary osteons (n = 323). Motic Images Plus 2.0 ML software (Motic China Group Co., Nanjing, China) was used to determine them. Cortical bone thickness was also evaluated. Twenty random areas were selected per thin section, and average thickness was calculated for each bone.

Atomic absorption spectrophotometry (Perkin Elmer 4100 ZL, Waltham, MA, USA) was used to determine the concentrations of Ca, magnesium (Mg), iron (Fe) and zinc (Zn) in the femoral bone. Briefly, bone samples were dried, digested in nitric acid, and diluted with distilled water [13].

Statistical analysis was conducted using SPSS Statistics 26.0 software. The data were expressed as mean ± standard deviation. Differences in all parameters examined were detected by ANOVA with Tukey’s post-hoc test. Statistical significance was assessed at *p* < 0.05.

## 3. Results

**Figure 1:**
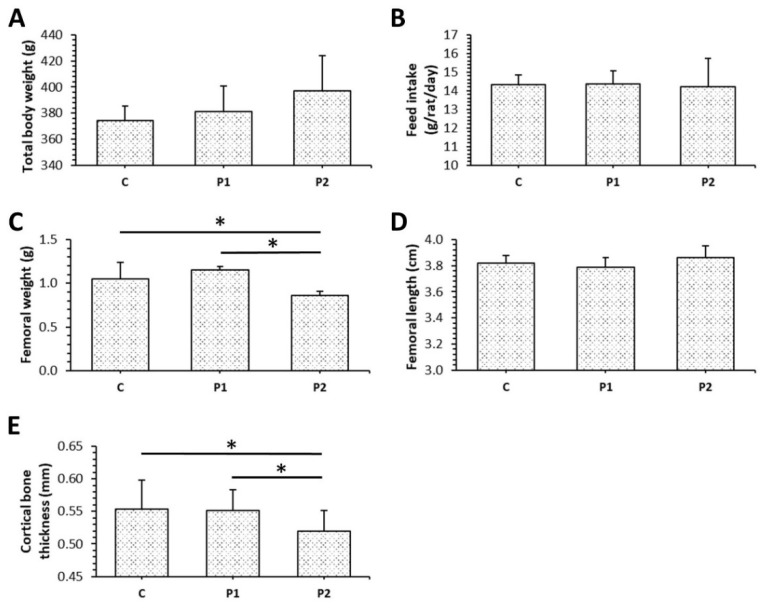
Total body weight (**A**), feed intake (**B**), femoral weight (**C**) and length (**D**), cortical bone thickness (**E**) in rats from C, P1, and P2 groups. * Significant differences (*p* < 0.05).

Total body weight (bw) of rats and femoral lengths were not affected by bee pollen supplementation, although an insignificant increase in body weight can be observed in both P1 and P2 groups. Feed intake in C group did not differ in comparison with P1 and P2 groups. On the other hand, significantly decreased femoral weight and cortical bone thickness were recorded in the P2 group versus the C group. Significant changes in the latter parameters mentioned above were also revealed between P1 and P2 groups (Figure 1A–D).

**Figure 2:**
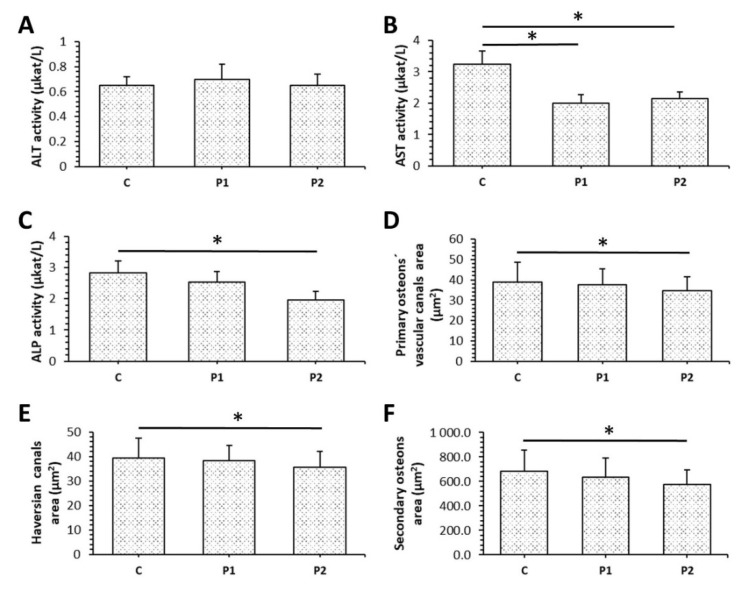
Levels of ALT (**A**), AST (**B**), ALP (**C**), and sizes of primary osteons’ vascular canals (**D**), Haversian canals (**E**), secondary osteons (**F**) in rats from C, P1, and P2 groups. * Significant differences (*p* < 0.05).

The levels of ALT did not vary between bee pollen supplemented groups and the control group. On the contrary, significantly lower values for AST were demonstrated in both P1 and P2 groups. In addition, ALP activity was significantly reduced in the P2 group when compared to the control (Figure 2A–C).

**Figure 3:**
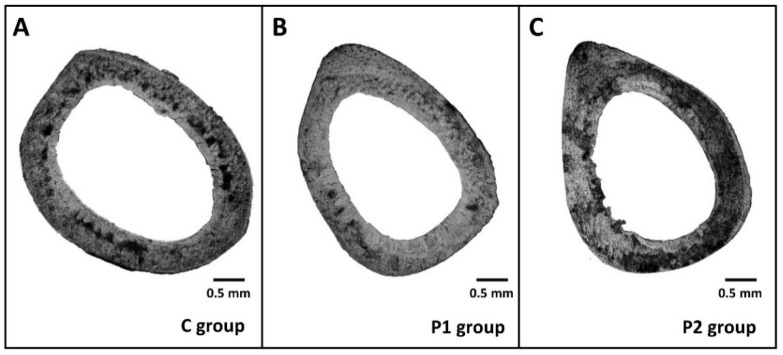
Cortical bone microscopic structure of rats from C (**A**), P1 (**B**) and P2 (**C**) groups.

Both surfaces (periosteal, endosteal) of the cortical bone were composed of non-vascular bone tissue in all groups studied. Primary vascular radial bone tissue was observed near the *endosteum* and in the middle part of *substantia compacta*. In the central part of the cortical bone, several secondary osteons have been identified in C, P1, and P2 groups. However, more primary and secondary osteons were recorded in *pars cranialis* (near periosteal border) in both bee pollen treated groups (Figure 3A−C).

Our results from histomorphological analysis of the cortical bone are summarized in Figure 2D–F. In the P2 group, a significantly decreased area of primary osteons’ vascular canals, Haversian canals, and secondary osteons was determined in comparison with the control. Lower doses of bee pollen did not influence sizes of aforementioned structures.

**Figure 4:**
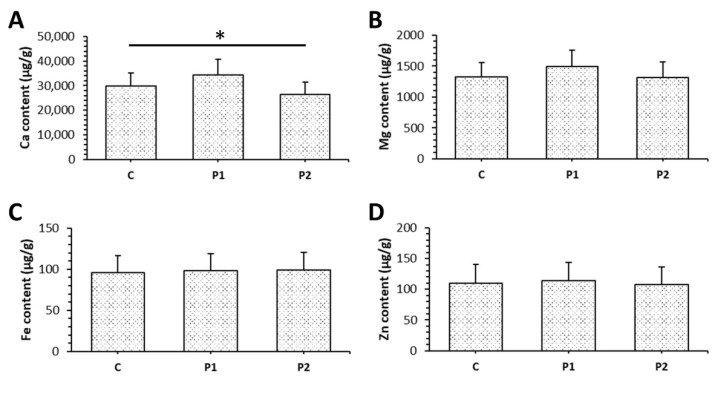
The content of Ca (**A**), Mg (**B**), Fe (**C**) and Zn (**D**) in femoral bones of rats from C, P1, and P2 groups. * Significant differences (*p* < 0.05).

The concentration of Ca in the femoral bone was significantly reduced in the P2 group against the C group (Figure 4A). Treatment with bee pollen did not affect the content of other mineral elements examined (Figure 4B–D).

## 4. Discussion

Bee pollen contains a lot of basic nutritional compounds important for productive, reproductive, and immune functions of animals. It has been used as a potential growth stimulator in many studies. According to Haro et al. [14], significantly increased total body weight was recorded in male rats receiving 1.0% of bee pollen for 10 days. The enhancement of final body weight was also observed in rabbits supplemented with 200 mg/kg bw of bee pollen for 5 weeks [15], those receiving 350 mg/kg bw of the pollen for 4 weeks [16], and also in broiler chickens treated with bee pollen at the level of 0.6% for 6 weeks [17]. On the other hand, no significant difference in total body weight was demonstrated between rats administered with bee pollen and their control in our study. Similarly, supplementation with bee pollen at the level of 0.2% (lasting 90 days) did not significantly influence body weight of rats [9]. Our findings suggest no differences in feed intake among all groups studied. However, Farag and El-Rayes [17] stated significantly decreased feed consumption in broiler chickens receiving bee pollen diets (0.2, 0.4 and 0.6% for 6 weeks) when compared to the control group. The dietary inclusion of bee pollen (200 mg/kg bw for 5 weeks) significantly reduced feed intake in rabbits [15]. It seems that longer administration with bee pollen leads to the elimination of significant changes in total body weight and feed intake between supplemented animals and their controls. Surprisingly, decreased femoral weight and cortical bone thickness were recorded in the P2 group. These results are partially consistent with those reported by Tomaszewska et al. [1], who noticed that 1.0% bee pollen administration (for 42 days) significantly reduced bone weight and mean relative bone thickness in Japanese quails, despite the lack of changes in their final body weight. Lower femoral weight was also determined in female rats receiving 0.75% of bee pollen for 90 days [10].

Dietary intake of bee pollen did not influence ALT activity in our study. However, significantly decreased values for AST were observed in both P1 and P2 groups. According to Attia et al. [15], levels of liver enzymes (AST, ALT) declined significantly in rabbits supplemented with bee pollen at a dose of 200 mg/kg bw. At the same time, Elnany and Elkholy [18] reported a reduction in AST, ALT values in rabbits treated with bee pollen (200, 300 and 400 mg/kg bw for 8 weeks). Treatment with bee pollen at the level of 0.6% (for 6 weeks) also decreased levels of both aforementioned enzymes in broiler chickens [17], thus indicating an ability of bee pollen to decline liver damage. Moreover, we determined a significantly reduced ALP activity in rats receiving 0.75% of bee pollen. The activity of ALP found in serum is a composite of isoenzymes, especially detected in liver and bone (more than 80%). Taking into account the bone, lower levels of ALP could be associated with Zn and Mg deficiency [19] and/or depressed osteoblast activity leading to a reduced bone formation [20]. Since no significant differences in Zn and Mg contents have been found between P2 and C groups, the decreased ALP activity indicated an inhibited bone formation rate in the P2 group. That evidence is also supported by significantly lower Ca concentration and reduced cortical bone thickness in those rats. In general, decreased cortical bone thickness is associated with increased stresses that could cause bone resorption [21], and simultaneously reduced Ca content in the femoral bone only confirms this fact. Our findings differ from those reported by Yamaguchi et al. [6], who mentioned a higher Ca concentration and increased ALP activity in the femoral bone of rats administered with bee pollen extract obtained from *Cistus ladaniferus* (5 and 10 mg/100 g bw). Yamaguchi et al. [22] also detected a stimulatory effect of bee pollen *Cistus ladaniferus* extract on bone formation and its inhibitory impact on bone resorption in vitro using osteoblast cells. However, Tomaszewska et al. [1] also stated a reduced mineralization in the tibia of Japanese quails supplemented with 1.0% of multifloral bee pollen. In addition, these bones were the weakest and have the greatest susceptibility to plastic deformation. Unlike previous studies, Kleczek et al. [7] and Oliveira et al. [8] reported no effect of bee pollen treatment (0.5% and 1.0% of feed) on Ca content and bone mineralization in broiler chickens. Rape bee pollen inclusion of 0.2% did not influence the concentration of Ca in the femoral bone of rats as well [9].

Higher number of primary and secondary osteons in *pars cranialis* of the cortical bone in both P1 and P2 groups might suggest an enhanced bone remodeling there. It is generally accepted that cranial and lateral sides contain fewer secondary osteons when compared to caudal and medial sides due to different tension and compression strains [23]. For this reason, we believe that enhanced density of secondary osteons in the cranial side could be associated with bee pollen treatment. On the other hand, significantly reduced sizes of primary osteons’ vascular canals, Haversian canals, and secondary osteons were demonstrated in rats after 0.75% bee pollen supplementation. This evidence might be consistent with a vasoconstriction of blood vessels in both canals and impaired blood flow. Generally, blood vessels supplying bones organize both bone remodeling and bone regeneration by delivering oxygen, nutrients, growth factors, or hormones to bone cells [24]. Decreased sizes of secondary osteons in rats from the P2 group could indicate a lower bone toughness. Significantly smaller osteons have been namely associated with decreased bone strength [25]. Interestingly, the lower dose (0.5%) of bee pollen did not affect sizes of aforementioned structures in the cortical bone of rats, similar to the treatment with bee pollen at the level of 0.2% in our previous study [9].

In summary, our results partially support the findings of Tomaszewska et al. [1], who also detected possible unfavorable impacts of bee pollen treatment on cortical bone properties using Japanese quails as an animal model. In terms of our study limitations, the number of rats used in the experiment was limited and only males were used. Therefore, it would be necessary to perform further research involving higher number of rats of both sexes. Additional analyses with different doses of bee pollen and longer duration of supplementation would also be valuable.

## 5. Conclusions

Our study revealed a dose-dependent impact of bee pollen supplementation on the femoral bone structure of rats. Several negative effects of bee pollen administration at the level of 0.75% on bone weight, cortical bone thickness, Ca content, ALP activity, sizes of primary osteons’ vascular canals, Haversian canals and secondary osteons have been determined, while the concentration of 0.5% had no effect. The results encourage caution in dosing and nutritional recommendations regarding bee pollen supplementation for preventive use. However, additional experiments are needed to verify the results obtained.
